# Effects of daidzein in regards to cytotoxicity *in vitro*, apoptosis, reactive oxygen species level, cell cycle arrest and the expression of caspase and Bcl-2 family proteins

**DOI:** 10.3892/or.2015.4133

**Published:** 2015-07-14

**Authors:** BING-JIE HAN, WEI LI, GUANG-BIN JIANG, SHANG-HAI LAI, CHENG ZHANG, CHUAN-CHUAN ZENG, YUN-JUN LIU

**Affiliations:** School of Pharmacy, Guangdong Pharmaceutical University, Guangzhou, Guangdong 510006, P.R. China

**Keywords:** daidzein, cytotoxicity *in vitro*, reactive oxygen species, mitochondrial membrane potential, western blot analysis

## Abstract

In the present study, the *in vitro* cytotoxicity of daidzein was evaluated in human BEL-7402, A549, HeLa, HepG-2 and MG-63 cancer cell lines. BEL-7402 cells were sensitive to daidzein treatment, with an IC_50_ value of 59.7±8.1 *µ*M. Daidzein showed no cytotoxic activity toward A549, HeLa, HepG-2 and MG-63 cells. Daidzein increased the levels of reactive oxygen species (ROS) and induced a decrease in mitochondrial membrane potential. Morphological and comet assays showed that daidzein effectively induced apoptosis in BEL-7402 cells. Additionally, daidzein caused cell cycle arrest at the G2/M phase in the BEL-7402 cell line. Daidzein downregulated the expression of Bcl-2, Bcl-x and Baid proteins and upregulated the levels of Bim protein in the BEL-7402 cells. The results demonstrated that daidzein induced BEL-7402 cell apoptosis through an ROS-mediated mitochondrial dysfunction pathway.

## Introduction

**Figure 1:**
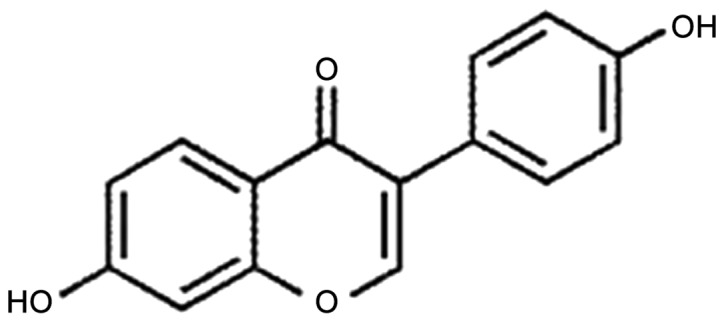
Structure of daidzein.

Consumption of soy-based foods has been associated with a lower risk of breast cancer, the most frequently diagnosed cancer in women worldwide (1,2). The isoflavones genistein and daidzein are naturally occurring phenolic compounds. The main sources of dietary isoflavones are soybeans, although they are present in many herbs and foods of botanical origin. Among the isoflavones, genistein is well-known to have a hypolipidemic effect and prevent cardiovascular disease by regulating lipid and carbohydrate metabolism (3,4). Daidzein has been demonstrated to be a potent antioxidant and has received much attention in relation to human health (5). In recent years, the anticancer activity of daidzein has been given much attention. Magee *et al* reported that daidzein suppressed MDA-MB-231 breast cancer cell invasion by reducing matrix metalloproteinase (MMP)-2 activity, suggesting an important role of daidzein in breast carcinogenesis (6). In another study, the inhibitory effect of daidzein on AML-1 cells was weak on day 3, yet it was evident on day 5 at concentrations of 20, 100, 200 and 400 *µ*M (7). In order to obtain more insight into the anticancer activity of daiazein (Fig. 1), in the present study, the cytotoxic effect of daiazein on BEL-7402, A549, HeLa, HepG-2 and MG-63 cell lines was investigated. Apoptosis, DNA damage, levels of reactive oxygen species and mitochondrial membrane potential in BEL-7402 cells was assessed by fluorescence microscopy. The percentage of apoptotic cells and cell cycle arrest were investigated by flow cytometry. The expression of Bcl-2 family proteins was performed by western blot analysis. The results demonstrated that daidzein induced BEL-7402 cell apoptosis through an ROS-mediated mitochondrial dysfunction pathway.

## Materials and methods

All reagents and solvents were purchased commercially and used without further purification unless otherwise noted. Ultrapure Milli-Q water was used in all experiments. Daiazein was purchased from the Aladdin Industrial Corporation. Dimethylsulfoxide (DMSO) and RPMI-1640 were purchased from Sigma. HepG-2 (human hepatocellular carcinoma), A549 (human lung carcinoma), BEL-7402 (hepatocellular), MG-63 (human osteosarcoma) and HeLa (human cervical cancer) cell lines were purchased from the American Type Culture Collection (ATCC; Manassas, VA, USA).

### Cytotoxic activity in vitro

3-(4,5-Dimethylthiazol-2-yl)-2,5-diphenyltetrazolium bromide (MTT) assay was used as previously described (8). Cells were placed in 96-well micro-assay culture plates (8×10^3^ cells/well) and grown overnight at 37°C in a 5% CO_2_ incubator. The compound tested was then added to the wells to achieve final concentrations ranging from 10^−6^ to 10^−4^ M. Control wells were prepared by addition of culture medium (100 *µ*l). The plates were then incubated at 37°C in a 5% CO_2_ incubator for 48 h. Upon completion of the incubation, stock MTT dye solution (20 *µ*l, 5 mg/ml) was added to each well. After 4 h, buffer (100 *µ*l) containing dimethylformamide (50%) and sodium dodecyl sulfate (20%) was added to solubilize the MTT formazan. The optical density of each well was measured with a microplate spectrophotometer at a wavelength of 490 nm. The IC_50_ values were determined by plotting the percentage of cell viability vs. concentration on a logarithmic graph and by reading the concentration at which 50% of cells remained viable relative to the control. Each experiment was repeated at least three times to obtain the mean values. Five different tumor cell lines were used in the present study: A549, BEL-7402, MG-63, HepG-2 and HeLa.

### Apoptosis assay by AO/EB staining method

BEL-7402 cells were seeded onto chamber slides in 6-well plates at a density of 2×10^5^ cells/well and incubated for 24 h. The cells were cultured in RPMI-1640 supplemented with 10% of fetal bovine serum (FBS) and incubated at 37°C in 5% CO_2_. The medium was removed and replaced with medium (final DMSO concentration, 0.05% v/v) containing daidzein (30 *µ*M) for 24 h. The medium was removed again, and the cells were washed with ice-cold phosphate-buffer saline (PBS), and fixed with formalin (4%, w/v). Cell nuclei were counterstained with acridine orange (AO) and ethidium bromide (EB) (AO, 100 *µ*g/ml; EB, 100 *µ*g/ml) for 10 min. The cells were observed and imaged with a fluorescence microscope (Nikon, Yokohama, Japan) with excitation at 350 nm and emission at 460 nm.

### Comet assay

DNA damage was investigated by means of comet assay. BEL-7402 cells in culture medium were incubated with 30 and 60 *µ*M of daidzein for 24 h at 37°C. The control cells were also incubated for the same time periods. The cells were harvested by a trypsinization process at 24 h. A total of 100 *µ*l of 0.5% normal agarose in PBS was dropped gently onto a fully frosted microslide, covered immediately with a coverslip, and then placed at 4°C for 10 min. The coverslip was removed after the gel had set. The cell suspension (50 *µ*l) (200 cells/*µ*l) was mixed with 50 *µ*l of 1% low melting agarose preserved at 37°C. A total of 100 *µ*l of this mixture was applied quickly on top of the gel, coated over the microslide, covered immediately with a coverslip, and then placed at 4°C for 10 min. The coverslip was again removed after the gel had set. A third coating of 50 *µ*l of 0.5% low melting agarose was placed on the gel and allowed to set at 4°C for 15 min. After solidification of the agarose, the coverslips were removed, and the slides were immersed in an ice-cold lysis solution (2.5 M NaCl, 100 mM EDTA, 10 mM Tris, 90 mM sodium sarcosinate, NaOH, pH 10, 1% Triton X-100 and 10% DMSO) and placed in a refrigerator at 4°C for 2 h. All of the above operations were performed under low lighting conditions to avoid additional DNA damage. The slides, after removal from the lysis solution, were placed horizontally in an electrophoresis chamber. The reservoirs were filled with an electrophoresis buffer (300 mM NaOH, 1.2 mM EDTA) until the slides were just immersed in it, and the DNA was allowed to unwind for 30 min in electrophoresis solution. Then the electrophoresis was carried out at 25 V and 300 mA for 20 min. After electrophoresis, the slides were removed, washed thrice in a neutralization buffer (400 mM Tris, HCl, pH 7.5). Cells were stained with 20 *µ*l of EB (20 *µ*g/ml^−^) in the dark for 20 min. The slides were washed in chilled distilled water for 10 min to neutralize the excess alkali, air-dried and scored for comets by fluorescence microscopy.

### Reactive oxygen species (ROS) detection

BEL-7402 cells were seeded into 6-well plates (Costar, Corning Inc., Corning, New York, NY, USA) at a density of 2×10^5^ cells/well and incubated for 24 h. The cells were cultured in RPMI-1640 medium supplemented with 10% of FBS and incubated at 37°C in 5% CO_2_. The medium was removed and replaced with medium (final DMSO concentration, 0.05% v/v) containing daidzein (30 *µ*M) for 24 h. The medium was removed again. The fluorescent dye 2′,7′-dichlorodihydrofluorescein diacetate (DCHF-DA; 10 *µ*M) was added to the medium to cover the cells. The treated cells were then washed with cold PBS-EDTA twice, collected by trypsinization and centrifugation at 1,500 rpm for 5 min. The cell pellets were then suspended in PBS-EDTA and imaged with a fluorescence microscope. The fluorescent intensity was determined by a microplate analyzer (Infinite M200; Tecan, Switzerland) with excitation at 488 nm and emission at 525 nm.

### Mitochondrial membrane potential assay

BEL-7402 cells were treated for 24 h with daidzein (30, 60 and 90 *µ*M) in 12-well plates and were then washed three times with cold PBS. The cells were detached with trypsin-EDTA solution. Collected cells were incubated for 20 min with 1 *µ*g/ml of JC-1 in culture medium at 37°C in the dark. Cells were immediately centrifuged to remove the supernatant. Cell pellets were suspended in PBS and imaged by fluorescence microscopy. The fluorescence intensity was determined by a microplate analyzer (Infinite M200) with excitation set at 488 nm and emission at 525 nm.

### Apoptosis assay by flow cytometry

After chemical treatment, 1×10^6^ cells were harvested, washed with PBS, then fixed with 70% ethanol and finally maintained at 4°C for at least 12 h. The pellets were stained with the fluorescent probe solution containing 50 mg/ml propidium iodide (PI) and 1 mg/ml Annexin in PBS on ice in dark for 15 min. Then the fluorescence emission was measured at 530 and 575 nm (or equivalent) using 488 nm excitation by a FACSCalibur flow cytometry (Becton, Dickinson and Company, Franklin Lakes, NJ, USA). A minimum of 10,000 cells were analyzed per sample.

### Cell cycle arrest by flow cytometry

BEL-7402 cells were seeded into 6-well plates (Costar, Corning Inc.) at a density of 2×10^5^ cells/well and incubated for 24 h. The cells were cultured in RPMI-1640 medium supplemented with 10% FBS and incubated at 37°C in 5% CO_2_. The medium was removed and replaced with medium (final DMSO concentration, 0.05% v/v) containing daidzein (30 *µ*M). After incubation for 24 h, the cell layer was trypsinized and washed with cold PBS and fixed with 70% ethanol. Twenty microliters of RNase (0.2 mg/ml) and 20 *µ*l of PI (0.02 mg/ml) were added to the cell suspensions and incubated at 37°C for 30 min. Then, the samples were analyzed with a FACSCalibur flow cytometry. The number of cells analyzed for each sample was 10,000 (9).

### Western blot analysis

BEL-7402 cells were seeded in 3.5-cm dishes for 24 h and incubated with different concentrations of daidzein in the presence of 10% FBS. The cells were harvested in lysis buffer. After sonication, the samples were centrifuged for 20 min at 13,000 g. The protein concentration of the supernatant was determined by BCA assay. Sodium dodecyl sulfate-polyacrylamide gel electrophoresis was carried out loading equal amount of proteins/lane. Gels were then transferred to polyvinylidene difluoride membranes (Millipore) and blocked with 5% non-fat milk in Tris-buffered saline with Tween-20 (TBST) buffer for 1 h. Then, the membranes were incubated with primary antibodies at a 1:5,000 dilution in 5% non-fat milk overnight at 4°C, and washed four times with TBST for a total of 30 min. The membranes were then incubated which the secondary antibodies conjugated with horseradish peroxidase at a 1:5,000 dilution for 1 h at room temperature and then washed four times with TBST. The blots were visualized with Amersham ECL Plus Western Blotting detection reagents according to the manufacturer's instructions. To assess the presence of comparable amount of proteins in each lane, the membranes were finally stripped to detect β-actin.

## Results and Discussion

### In vitro cytotoxicity assay

The cytotoxicity *in vitro* of daidzein was evaluated by the MTT method in 5 cancer cell lines: BEL-7402, A549, HeLa, HepG-2 and MG-63. The IC_50_ values of daidzein against the selected cell lines are listed in Table I. After the BEL-7402, A549, HeLa, HepG-2 and MG-63 cells were exposed to 6.25, 12.5, 25, 50 and 100 *µ*M of daidzein for 48 h, the IC_50_ value of daidzein toward BEL-7402 cells was 59.7±8.1 *µ*M. Obviously, daidzein showed moderate cytotoxic activity against the BEL-7402 cells. Unexpectedly, daidzein had no cytotoxic activity against the A549, HeLa, HepG-2 and MG-63 cells; the IC_50_ values were >100 *µ*M. The results demonstrated that daidzein displays different cytotoxic effects on different cancer cell lines.

### Apoptosis assay with AO/EB staining method

**Figure 2:**
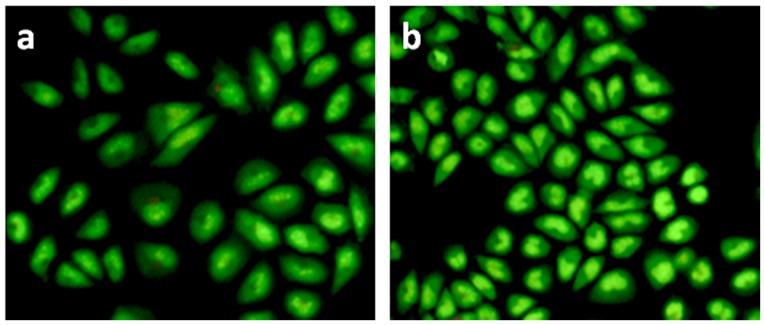
Apoptosis in (a) control BEL-7402 cells and (b) BEL-7402 cells exposed to 30 *µ*M of daidzein for 24 h, and stained with AO and EB. AO, acridine orange; EB, ethidium bromide.

Induction of apoptosis is one of the considerations in drug development, as most cytotoxic anticancer drugs in current use induce apoptosis in susceptible cells (10). In order to determine whether or not daidzein induces chromatin condensation and fragmentation, both of which are recognized morphological features of apoptosis, BEL-7402 cells were treated with 30 *µ*M of daidzein for 24 h. As shown in Fig. 2a, control BEL-7402 cells were stained with uniform green fluorescence and no apoptotic features were observed. Following treatment of BEL-7402 cells with daidzein for 24 h, obvious morphological changes and green apoptotic cells containing apoptotic characteristics such as cell blebbing, nuclear shrinkage and chromatin condensation were observed (Fig. 2b). The results suggest that daidzein induced BEL-7402 cell apoptosis.

### DNA damage assay

**Figure 3:**
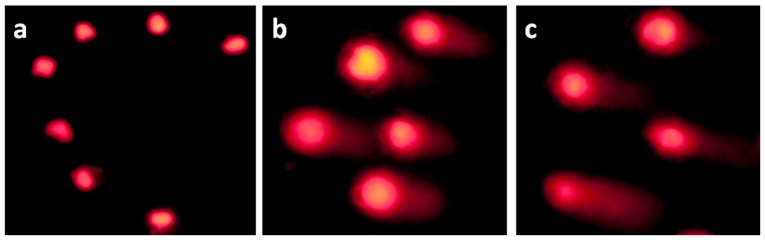
Comet assay of (a) control BEL-7402 cells and BEL-7402 cells exposed to (b) 30 and (c) 60 *µ*M of daidzein for 24 h folowing staining with EB. EB, ethidium bromide.

DNA fragmentation is a hallmark of apoptosis, mitotic catastrophe, or both (11). DNA damage is assayed by the use of single-cell gel electrophoresis (comet assay) in agarose gel matrix. As shown in Fig. 3a, in the control cells, no comet like appearance was observed. After BEL-7402 cells were exposed to 30 (Fig. 3b) and 60 *µ*M (Fig. 3c) of daidzein for 24 h, a statistically significant number of well-formed comets were noted. Furthermore, the length of the comet tails increased with increasing concentrations of daidzein. These results indicate that daidzein induced DNA fragmentation, which was further evidence of apoptosis.

### Detection of ROS levels by fluorescence microscope

**Figure 4:**
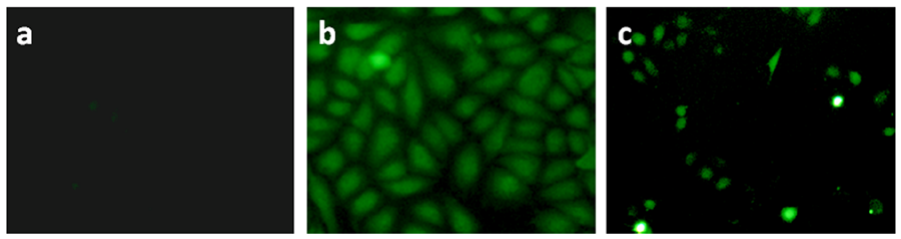
Intracellular ROS was detected in (a) control BEL-7402 cells and BEL-7402 cells exposed to (b) Rosup and (c) 30 *µ*M of daidzein, for 24 h. Rosup was used as a positive control. ROS, reactive oxygen species.

To determine the effect of daidzein on intracellular ROS generation, DCHF-DA was used as a fluorescent probe. DCFH-DA is a fluorescent dye that diffuses through cell membranes and is hydrolyzed by intracellular esterases to DCFH. In the presence of ROS, DCFH is oxidized to DCF, which is fluorescent and its level corresponds to the level of generated ROS. As shown in Fig. 4a, in the control, no obvious fluorescence images were found. Following treatment of BEL-7402 cells with Rosup (Fig. 4b, positive control) and 30 *µ*M of daidzein (Fig. 4c) for 24 h, the bright green fluorescence images were observed. The results indicate that daidzein increased the levels of ROS.

### Changes in the mitochondrial membrane potential

**Figure 5:**
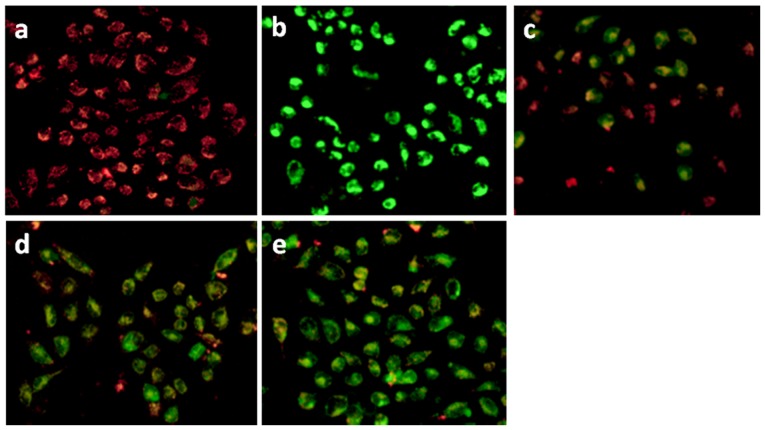
Mitochondrial membrane potential of BEL-7402 cells was determined with JC-1 as a fluorescence probe staining method. (a) Control BEL-7402 cells and cells exposed to (b) cccp and (c) 30, (d) 60 and (e) 90 *µ*M of daidzein for 24 h. cccp was used as a positive control.

The changes in the mitochondrial membrane potential were determined by fluorescence microscope. JC-1 was used as a fluorescence probe for detecting the changes in the mitochondrial membrane potential induced by daidzein. JC-1 forms aggregates, which have a red fluorescence emission peak at high mitochondrial membrane potential; JC-1 forms monomers, which emit a green fluorescence peak at low mitochondrial membrane potential. As shown in Fig. 5a, in the control, JC-1 exhibited a red fluorescence (JC-1 aggregates) confirming high mitochondrial membrane potential. After BEL-7402 cells were exposed to cccp (Fig. 5b) and 30 (Fig. 5c), 60 (Fig. 5d) and 90 *µ*M (Fig. 5e) daidzein for 24 h, JC-1 showed green fluorescence (JC-1 monomers) with little red fluorescence corresponding to low mitochondrial membrane potential. The changes from red to green fluorescence indicate a decrease in mitochondrial membrane potential. Moreover, the green fluorescence increased and the red fluorescence decreased with increasing concentrations of daidzein. The results demonstrated that daidzein induced a decrease in mitochondrial membrane potential and daidzein induced apoptosis in the BEL-7402 cells through a mitochondrial signal transduction pathway.

### Apoptosis assay by flow cytometry

**Figure 6:**
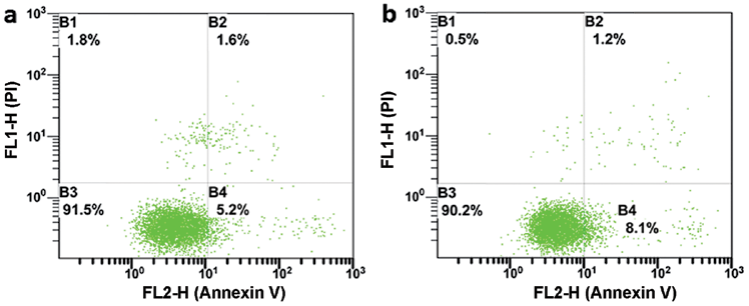
Apoptosis of (a) control BEL-7402 cells and (b) BEL-7402 cells exposed to 30 *µ*M of daidzein for 24 h.

AO/EB staining studies showed that daidzein induced the apoptosis of BEL-7402 cells. To determine the percentage of apoptotic and necrotic BEL-7402 cells, the cells were treated with 30 *µ*M of daidzein for 24 h and stained with Annexin V and PI followed by cell apoptosis analyses using flow cytometry. The percentages of apoptotic, necrotic and BEL-7402 living cells are shown in Fig. 6. In the control cells (Fig. 6a), the percentage of apoptotic (B4) and necrotic (B2) cells were 5.2 and 1.6%, respectively. Following treatment of BEL-7402 cells with daidzein for 24 h (Fig. 6b), the percentages of apoptotic and necrotic cells were 8.1 and 1.2%, respectively. The increase in the percentage of apoptotic cells of 2.9% suggests that daidzein induced apoptosis in the BEL-7402 cells.

### Cell cycle arrest studies

**Figure 7:**
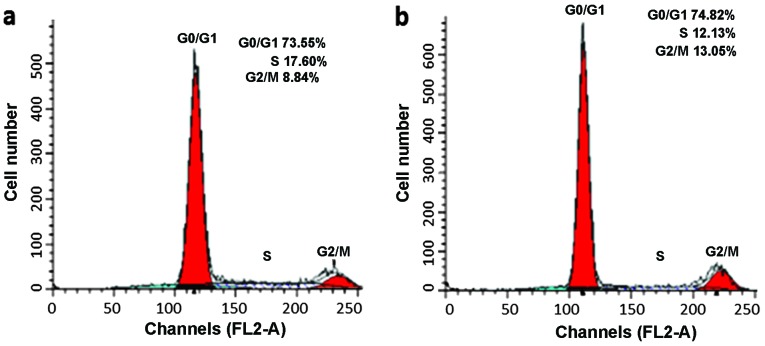
Cell cycle distribution of he (a) control BEL-7402 cells and (b) BEL-7402 cells following treatment with 30 *µ*M of daidzein for 24 h.

Flow cytometry was used to determine the effects of daidzein on the cell cycle progression of BEL-7402 cells. As shown in Fig. 7a, in the control cells, the percentage of cells in the G2/M and S phases were 8.84 and 17.60%, respectively. After BEL-7402 cells were exposured to 30 *µ*M of daidzein (Fig. 7b) for 24 h, the percentage of cells at the G2/M and S phases were 13.05 and 12.13%, respectively. An evident increase of 4.21% in the percentage of cells at the G2/M phase was observed, which was accompanied by a corresponding reduction of 5.47% in the percentage of cells in the S phase. The data showed that daidzein induced cell cycle arrest at the G2/M phase.

### Expression of caspase and Bcl-2 family proteins

**Figure 8:**
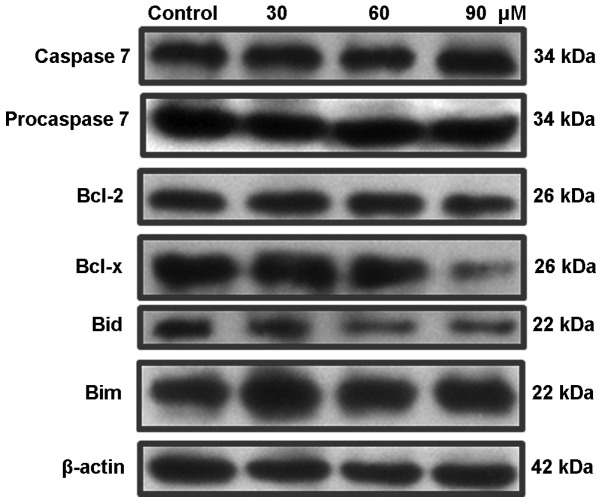
Western blot analysis of caspase 7, procaspase 7, Bcl-2, Bcl-x Bid and Bim in BEL-7402 cells treated with different concentrations of daidzein for 24 h. β-actin was used as an internal control.

Caspase 3 and 7 are executioners of apoptosis as the processing of their substrates leads to morphological changes associated with apoptosis, including DNA degradation, chromatin condensation and membrane blebbing (12). The expression of caspase 7 and procaspase 7 was assayed by western blot analysis. As shown in Fig. 8, after the treatment of BEL-7402 cells with different concentrations of daidzein, the expression levels of caspase 7 increased, whereas the expression levels of procaspase 7 were downregulated. To investigate the effect of daidzein on the expression levels of antiapoptotic and proapoptotic proteins, BEL-7402 cells were treated with different concentrations of daidzein for 24 h. As expected, the expression levels of antiapoptotic proteins Bcl-2 and Bcl-x were downregulated. The levels of the proapoptotic protein Bid decreased, whereas the expression of Bim was upregulated. These results demonstrate that daidzein induced apoptosis in BEL-7402 cells through activation of caspase 7, downregulation of Bcl-2, Bcl-x and Bid, upregulation of Bim and ROS-mediated mitochondrial dysfunction pathways.

In conclusion, daidzein showed moderate cytotoxic activity in BEL-7402 cells, while no cytotoxicity toward HeLa, A549 and MG-63 cells was noted. The compound induced the apoptosis of BEL-7402 cells. Daidzein increased the levels of ROS and induced a decrease in mitochondrial membrane potential. Additionally, daidzein inhibited the cell cycle progression of BEL-7402 cells at the G2/M phase. The compound activated caspase 7, downregulated the expression of Bcl-2, Bcl-x and Bid, and upregulated the expression of Bim. In summary, daidzein induced apoptosis in the BEL-7402 cells by an ROS-mediated mitochondrial dysfunction pathway, which was accompanied by regulation of Bcl-2 family proteins.

## Figures and Tables

**Table I:** IC_50_ values of daidzein in the BEL-7402, HeLa, A549, HepG-2 and MG-63 cell lines.

Compound	IC_50_ (*µ*M) in cell lines				
	BEL-7402	HeLa	A549	HepG-2	MG-63
Daidzein	59.7±8.1	97.9±11.3	>100	>100	>100
